# Combined Effect of Chia, Quinoa and Amaranth Incorporation on the Physico-Chemical, Nutritional and Functional Quality of Fresh Bread

**DOI:** 10.3390/foods9121859

**Published:** 2020-12-12

**Authors:** Karla Carmen Miranda-Ramos, Claudia Monika Haros

**Affiliations:** 1 Faculty of Chemical Engineering, University of Guayaquil, Cdla. Universitaria Av. Delta y Av. Kennedy, Guayaquil 090514, Ecuador; karla.mirandara@ug.edu.ec; 2 Institute of Agrochemistry and Food Technology (IATA-CSIC), 46980 Valencia, Spain

**Keywords:** bread, *Salvia hispanica* L., *Chenopodium quinoa* Willd, *Amaranthus caudatus*, technological characteristics, nutritional value

## Abstract

With regard to constant technological innovations in the bakery sector in order to increase bread nutritional value without affecting its technological and sensory characteristics, we applied pseudocereals/oilseeds to obtain an optimal formulation. A factorial design 3^3^ was used and the independent factors were chia flour (levels: 0, 10, 20% flour basis), quinoa flour (levels: 0, 20, 40% flour basis), and amaranth flour (levels: 0, 20, 40% flour basis). Their effects and interactions were studied through the response surface methodology to optimise the bread formulation from a holistic viewpoint, which included the nutritional, technological and sensory characteristics. The optimum formulation with the highest quality was the blend made with 10, 4, and 20% of chia, quinoa, and amaranth, respectively. The results showed a significant increase in protein amount, ash, lipids, and crumb firmness compared to wheat bread. The calorie value of the control sample and the optimised formula were significantly similar, bearing in mind the high lipid amounts present in raw materials. Loaf-specific volume slightly decreased in comparison to control bread, as expected in formulations with gluten-free raw materials and a large amount of fibre. The optimised formula presented nutritionally/functionally higher indexes and similar overall acceptability to the control bread (*p* < 0.05).

## 1. Introduction

In the last few years, scientific studies have demonstrated that the regular intake of wholemeal or whole grain products prevents certain chronic diseases from developing, such as cardiovascular diseases, type 2 diabetes, and certain cancer types. Hence, consumer interest in such products has grown, although consumer acceptability is conditioned by their sensorial aspects despite being nutritional food with biological functionality in our organism [1,2]. Thanks to efforts to develop healthy and appealing bread products to supply nutritional, technological, and sensorial quality requirements, researchers have studied different strategies in order to develop products that use wholemeal flours with coadjuvants/additives that cover these requirements. These include adding baking enhancers, such as enzymes and/or chemical compounds [1,3,4], using wholemeal flours with different granulometries to increase sensorial quality [5,6,7], and/or partially replacing flour with more nutritional and healthy ingredients such as legumes [8,9,10], oilseeds [11,12,13], and pseudocereals [14,15,16]. The use of wholemeal flours of legumes, oilseeds, cereals, and pseudocereals increases their mineral content, but this increase comes with higher levels of phytic acid (Ins*P*_6_), forming insoluble compounds that inhibit their bioavailability. Some strategies can increase the bioavailability of minerals when using sourdough [17,18], or exogenous phytases, which are bread fermentation starters that produce phytases [4,15], or chemical agents such as ferric sodium ethylene diamine tetra acetic acid [3], among other strategies.

Consequently, the industry in this sector has closely examined the strategy of substituting refined wheat flour for wholemeal ingredients with high added value, such as pseudocereals, legumes, and/or oilseed so that more wholemeal foods offering better technological properties are eaten (loaf-specific volume, and crumb and crust colour and texture) with better nutritional properties (better amino acid and lipid profile, higher mineral content, better protein digestibility, and less starch digestibility) [19]. According to a considerable number of studies, baking products supplemented with wholemeal quinoa, amaranth, or chia flours have a higher nutritional value, but the end product’s technological and sensorial quality is lost [12,15,20,21,22]. Generally speaking, loss of quality with formulations enriched with different ingredients to wheat is due to gluten dilution, which affects all the bread-making process steps in accordance with the substitution level and the ingredient in question [23]. Although quality is compromised with such products, their nutritional value increases. This occurs in the bread formulations replaced with quinoa wholemeal flour, which not only contributes to daily diet fibre intake and daily Fe and Zn requirements, but also improves the ω-6/ω-3 ratio and protein quality [14,15]. Nevertheless, loaf-specific volume is reduced with textural changes in crumbs such as crumb firmness; crumb grain with bigger pores and thin walls; low resilience, cohesion, and elasticity; and a bitter taste [24,25]. The behaviour of the baking products replaced partially with different amaranth species was similar [16].

Replacing wheat flour with chia did not lead to loss of end product quality, and consumer acceptability was higher [11]. However, this tendency did no remain when the level of this oilseed was raised, taking in account the current EU regulations of a maximum level of 10% in bakery products, European Food Safety Authority [26]. Regarding the nutritional profile, as with bread products made with pseudocereals, bread made with chia contained more minerals, lipids, dietary fibre, and proteins, all with a higher biological value. Including chia in products results in lower glycaemic index (GI) and better saturated fatty acid (SFA)/polyunsaturated fatty acid (PUFA) ratios [12].

Hence, the main objective of this study was to develop top quality bread by substituting wheat flour for an optimum mixture of wholemeal quinoa, amaranth, and chia flours to maximise its physico-chemical, technological, nutritional, and sensorial properties by a factorial design 3^3^ and by following the response surface methodology (RSM). Another aim was to evaluate the nutritional value of the optimised formulation by considering its contribution to the daily recommended intake of fatty acids (omega), minerals (Ca, Fe, Zn), and dietary fibre (soluble and insoluble) and its protein quality, and to estimate its glycaemic index (GI) values in vitro. 

## 2. Materials and Methods

### 2.1. Materials

Quinoa (*Chenopodium quinoa* Willd), black chia (*Salvia hispanica* L.), and amaranth (*Amaranth caudatus*) flour (Inca’s treasure, Quito, Ecuador) were milled in a hammer type cyclone mill and at standard sieve (0.8 mm) (Lab Mill 3100, Perten Instruments, Huddinge, Sweden) and stored at 14 °C. Dehydrated yeast (*Saccharomyces cerevisiae*, Maizena, Spain) was used as a starter. Commercial wheat flour and whole wheat flour obtained from HARINERA LA META S.A. (part of La Meta Group, the Vall Companys Group’s flour division, Barcelona, Spain) was employed for the bread-making process.

### 2.2. Bread-Making Procedure

The control bread dough formula consisted of wheat flour (300 g), compressed yeast (3% flour basis), sodium salt (1.6% flour basis), and distilled water (up to optimum absorption, 500 Brabender Units). The 27 bread formulations with amaranth, quinoa, and/or chia, obtained by factorial design 3^3^, were mixed for 7 min, left for 10 min, divided (100 g), kneaded, and then left again (15 min). Dough was manually rolled, proven (up to optimum volume increase at 28 °C, 85% relative humidity), and baked at 180 °C/29 min. Temperature and volume increase of dough was monitored at regular intervals during fermentation. After fermentation, dough was baked in an electric oven and cooled at room temperature for 60 min for subsequent analyses.

### 2.3. Composition of Flours and Bread

Proximate analyses of raw materials and breads were performed in terms of moisture, total dietary fibre (TDF), and starch according to the approved Association of Official Agricultural Chemistry 925.09, 991.43, and 996.11, respectively [27]. Protein determination was carried out by the Dumas combustion method and a nitrogen conversion factor: 5.7/wheat flour; 5.53/quinoa, amaranth, chia whole flours; 5.83/wheat wholemeal; and 6.25/breads according to ISO (International Organization for Standardization)/TS (Technical Specification) 16634-1 and ISO/TS 16634-2 [28]. Lipid and ash contents were established according to Official Methods 30-10 and 08-03, respectively, from the American Association of Cereal Chemists [29]. Measurements were taken in triplicate.

### 2.4. Technological Parameters

The analysed technological parameters were as follows: loaf-specific volume (cm^3^/g) by measuring volume (cm^3^) by seed displacement (volume-meter, Chopin, France) and weight (g), the width/height ratio of the central slice (cm/cm), and colour tristimulus parameters (Chromameter CR-400, Konika Minolta Sensing, Japan). From the colour parameters, we calculated the total colour difference (ΔE*) by the Equation (1): Samples were analysed at least in triplicate [11].

ΔE* = [(ΔL*)^2^ + (Δa*)^2^ + (Δb*)^2^]^1/2^ (1)

Crumb texture was determined by the texture profile analysis using a TA-XT Plus Texture Analyser (Stable Micro Systems, Godalming, United Kingdom). A 2 cm-thick slice of bread was compressed twice by a stainless steel 0.5 cm diameter plunger, moving 1.0 min/s to a penetration distance of 50%, with an interval of 50 s between compressions. The following parameters were evaluated: firmness, springiness, cohesiveness, and chewiness.

The digital image analysis was used to measure bread crumb structure. Digital images were taken by an EVOCAM-II Macroscope (Vision engineering, Woking, United Kingdom). Images were processed and analysed by the Nis Elements BR 3.2 software (Nikon Corporation, Japan) and also Fiji (ImageJ 1.49q Software, National Institutes of Health, Bethesda, MD, USA). A single 10 × 10 mm square field of view of two central slices (10 mm thick) of both loaves was used to yield three digital images per treatment. Data were processed using the Statgraphics Plus 16.1.03 software (Bitstream, Cambridge, MN, USA). The chosen crumb grain features were cell area/total area, cm^2^/cm^2^; wall area/total area, cm^2^/cm^2^; number of cells per cm^2^; and mean cell area, mm^2^.

### 2.5. Fatty Acid Profile

Samples were transesterified to convert triglycerides into fatty acid methyl esters (FAMEs), following the methodology previously described by the American Oil Chemists’ Society [30]. The fatty acid composition and quantification were determined by gas chromatography with a capillary column (SP 2330 on 100/120 WAW-60 m × 0.25 mm × 0.5 μm) and a flame ionisation detector according to the International Union of Pure and Applied Chemistry Method 2.302 [31]. Measurements were taken in triplicate.

### 2.6. Mineral Composition

The total Ca, Fe, and Zn concentrations were determined in a flame absorption spectrometer at the Analysis of Soils, Plants and Water Service of the Institute of Agricultural Sciences, Madrid (Spain). Each sample (0.5 g) was placed in a Teflon perfluoroalkoxy vessel and digested with HNO_3_ (4 mL, 14 M) and H_2_O_2_ (1 mL, 30% *v*/*v*) attack. Samples were irradiated at 800 W (15 min at 180 °C) by a Microwave Accelerated Reaction System (MARS, Charlotte, NC, USA). At the end of the digestion programme, the digest was placed in a polypropylene tube and made up to final volume with distilled water. Measurements were taken in triplicate [12].

### 2.7. Determination of Myo-Inositol Hexakisphosphate

The *myo*-inositol hexakisphosphate or phytic acid (Ins*P*_6_) present in raw materials and the residual in the bread formulations after the bread-making process was measured as phosphorus released by phytase and alkaline phosphatase by a simple quantity K-PHYT method [32]. This method consists of acid extraction of phytates, followed by treatment with phytase and alkaline phosphatase enzymes to release phosphates from the *myo*-inositol ring. The total released phosphate was measured by a colorimetric technique according to the AOAC method 986.11 [27]. Samples were analysed in triplicate.

### 2.8. Amino Acids Profile

For the amino acid analysis, 10 mL of hydrolysed sample was prepared with 4 mL of 6 N HCl. Solutions were capped in a nitrogen atmosphere for 24 h. Amino acids were determined by acid hydrolysis after derivatisation with diethyl ethoxymethylenemalonate in a high-performance liquid chromatography (HPLC) Model 600E multisystem with a 484 UV–VIS detector (300 mm × 3.9 mm) and a reversed-phase column (Novapack C18, 4 m; Waters), acetonitrile in the binary gradient, detection at 280 nm, with D,L-α-aminobutyric acid as the internal standard. Solvents were injected into the column at a flow rate of 0.9 mL/min. Temperature remained at 18 °C [33].

### 2.9. In Vitro Protein Digestibility, Essential Amino Acids, and Nutritional Index

The in vitro gastric digestion of bread samples was carried out according to the methodology described by Sanz-Penella et al. [18]. The dry and ground of bread was subjected to a simulated gastrointestinal digestion, beginning by a simple digestion with the addition of pepsin (800–2500 Units/mg protein), pancreatin (activity, 4×; United States Pharmacopeia (USP)/reference standard specifications), and bile extract, which were demineralised with Chelex-100 before use. Briefly, 6 mL of an isotonic saline solution (140 mM NaCl, 5 mM KCl) was added to the sample breads (1.000 ± 0.001 g), and mixtures were acidified to pH 3.0 with 0.1 mol/L HCl. Then, 0.96 mL of a pepsin solution (0.01 g/mL) was added, and the mixture was incubated for 1 h at 37 °C (gastric digestion). Later, the protein contained in the gastric digestion solution was measured by the Bradford method with bovine serum albumin as the standard. 

The essential amino acid index (EAAI) was calculated according to Motta et al. [34] by applying the following Equation (2):

(2) $$EAA=0.1\left[log\left(\frac{a_{1}}{a_{1s}}\times 100\right)\;+log\left(\frac{a_{2}}{a_{2s}}\times \;100\right)+\ldots log\left(\frac{a_{n}}{a_{ns}}\right)\times 100\right]$$

where *a*_1_, *a*_2_, ..., *a_n_* are the amino acid contents in the sample, and *a*_1*s*_, *a*_2*s*_, …, *a_ns_* are the essential amino acid requirements in the protein standard [35].

The nutritional index (NI, Equation (3)) normalises the qualitative and quantitative variations of the test protein compared to its nutritional status. The NI was calculated by the equation of Crisan and Sands [36], which considers all the factors to be of equal importance:

(3) $$NI=\frac{EAAI\;Protein\;\left(\%\right)}{100}$$

### 2.10. In Vitro Glycaemic Index Estimation

To evaluate the in vitro rate of starch hydrolysis, we followed the method described by Goni et al. [37] with slight modifications according to Sanz-Penella et al. [4]. The hydrolysis index (HI) was calculated from the area under the curve (AUC) from 0 to 120 min for samples as a percentage of the corresponding area of reference (wheat bread) (HI = AUC sample/AUC wheat bread × 100). The glycaemic index (GI) was calculated by the equation GI = 0.549 × HI + 39.71. Measurements were taken in triplicate. The predicted glycaemic load (pGL) was calculated for a 100 g bread portion from the glucose-related GI according to pGL = glycaemic index × total carbohydrates/100, and by taking into account the total carbohydrates of each sample [38].

### 2.11. Preliminary Sensory Evaluation

The parameters measured in the control and optimised bread formulae were appearance, texture, taste, and overall acceptability, evaluated by a panel of 50 untrained tasters who usually purchase wheat bread using a 9-point hedonic scale of global acceptance: (9) “Especially like”; (8) “Very much like”; (7) “Moderately like”; (6) “Somewhat like”; (5) “Neither like nor dislike”; (4) “Slightly dislike”; (3) “Moderately dislike”; (2) “Very much dislike”; (1) “Especially dislike” [15].

### 2.12. Factorial Design

In order to study the effect of replacing wheat flour with nutritious ingredients on the physico-chemical, nutritional, technological, and sensory properties, we used a factorial design 3^3^. The 3 studied factors were the percentage of wheat flour replacement with whole chia flour at 3 levels (0, 10, and 20%), whole quinoa flour at 3 levels (0, 20, and 40%), and amaranth flour at 3 levels (0, 20, and 40%). The run conditions of the factorial design in terms of the experimental conditions and coded values are shown in Table 1.

The design enabled us to approximate the experimental data $\left(Y_{obs}\right)$ with a response surface model expressed as coded values according the Equation (4):

(4) $$Y_{obs}=a_{0}+a_{1}x_{1}+a_{2}x_{2}+a_{3}x_{3}+a_{11}x_{1}^{2}+a_{12}x_{1}x_{2}+\;a_{13}x_{1}x_{3}+a_{22}x_{2}^{2}+a_{23}x_{2}x_{3}+\;a_{33}x_{3}^{2}+\;\varepsilon$$

where $x_{1}$ is the design factor whole chia flour; $x_{2}$ is the whole quinoa flour; $x_{3}$ is whole amaranth flour; and coefficients $a_{1}$, $a_{2}$, and $a_{3}$ are the main effects of $x_{1}$, $x_{2}$, and $x_{3}$, respectively. The square coefficients $\left(a_{ii}\right)$ indicate whether any of the variables have a maximum or minimum in the experimental domain, whereas the mixed coefficients $\left(a_{12}\right)$, $\left(a_{13}\right)$, and $\left(a_{23}\right)$ represent interactions between factors. The difference between the experimental data $\left(Y_{obs}\right)$ and model $Y_{calc}$ gives the residual (ε). For each response, the RS-Q (squared correlation coefficient) was calculated, which is the fraction of variation of the response explained by the model.

The response variables were content of lipids, ash, proteins, and calorie value of bread (nutritional characteristics); piece-specific volume, crumb and crust colour, crumb firmness, and shape ratio (technological qualities); and sensory evaluation, such as the product’s appearance, texture, taste, and overall consumer acceptability. Twenty-seven formulations were studied with different proportions of amaranth, quinoa, and/or chia, as shown in Table 1.

### 2.13. Statistical Analysis

One-way ANOVA and Fisher’s least significant differences (LSD) were applied to establish significant differences between samples (*p* < 0.05). Homogeneity of variances was tested using Levene’s tests and normally distributed on the basis of the Shapiro–Wilk test. All statistical analyses and optimisation of multiple responses were carried out with the Statgraphics Plus 16.1.03 software (Bitstream, Cambridge, MN, USA).

The desired objective was selected for each dependent variable on the basis of the values obtained by the control condition (wheat bread). For the numerical optimisation, all the independent variables were left within their predetermined range, while the dependent variables were optimised by taking into account the nutritional, technological, and sensory aspects.

## 3. Results and Discussion

### 3.1. Flour Composition

The chemical composition of the flours herein employed is found in Table 2. The protein content of the quinoa, amaranth, and chia flours was significantly higher than the protein content of both the refined wheat and commercial wholemeal wheat flours. The protein contents of amaranth, quinoa, and chia in this study fell in line with those previously reported by other researchers: 13.1–21.5%, 8.0–22.0%, and 18.2–25.3%, respectively [39,40,41]. Variations in protein content and the amino acid profile depend on growth conditions and genotype [42]. This means that wholemeal flours from the crops of Andean origin used in the present study could cover the protein requirements recommended by both the Food and Agriculture Organisation (FAO)/World Health Organisation (WHO), which stress chia protein [12,43]. The high lysine content in the amino acid profile is present in quinoa, amaranth, and chia grains, whereas is deficient in cereals [41,43,44]. In grains, proteins are distributed heterogeneously, and have a different biological quality depending on grain parts. For instance, in amaranth, 65% of lysine-rich proteins are found in the germ, but only 35% of lysine-poor protein lie in the endosperm. The exact opposite occurs in cereals where 85% of lysine-poor protein appears in the endosperm [45]. This is why it is so important to eat food made with wholemeal flours because they supply much nutrition, including protein quality and quantity.

Lipid content in the quinoa, amaranth, and chia flours was significantly higher than the lipid content in refined wheat flours because the germ is removed during the refining process, as are outer bran layers, which is where the biggest quantity of fat and fibre is found [46]. Nonetheless, the lipid content of chia seeds of Andean origin can vary according to agronomic conditions (30.7–41.5%) [12,47]. The high percentage of lipids in wholemeal quinoa and amaranth flours is because the germ remains after milling whole grains. These results fall in line with those found in the literature, lying between 2% and 11% in quinoa, and between 5.6% and 19.3% in amaranth [40], and are similar to the results obtained for wholemeal wheat flour (1–2.5%) [48].

Variation in lipid content is generally due to inter-species differences, environmental factors, and crop-growing practices [12,43,49]. The quality of the lipids present in the herein used flours is characterised by their high content of PUFA and their suitable linoleic acid (LA)/alpha-linolenic acid (ALA) ratios, whose low values positively impact health. The lowest ratio of the employed ingredients was found for chia for its high alpha-linolenic acid content (50–57%), followed by quinoa, then amaranth, and finally by wheat [40]. Thus, employing these crops could help to promote healthy eating and prevent cardiovascular diseases [47]. 

Total fibre content was significantly higher in the chia flour compared to the wholemeal quinoa, amaranth, and wheat flours (Table 2). Of the total chia fibre, 17% corresponded to soluble dietary fibre (SDF) and the rest to insoluble dietary fibre (IDF), which agrees with values reported in the literature (7–15%/SDF; 85–93%/IDF) [43,50]. No significant differences were found between total fibre content for quinoa and amaranth, which was higher than in wheat. The same trend was observed with both soluble and insoluble fibre, which also agrees with the values reported in the literature for soluble fibre content in quinoa and amaranth versus wheat. Moreover, the percentage ratio between soluble and insoluble fibre, compared to total fibre, in the wholemeal quinoa (29%/SDF; 71%/IDF) and amaranth (20%/SDF; 80%/IDF) flours were similar to those shown in the literature [20,41].

Generally speaking, the wholemeal flours made with ancient crops presented a significantly higher ash concentration, which was directly related to a higher mineral content, where chia stood out, followed by quinoa and amaranth, and finally by wheat (Table 2). Regarding starch content, this main carbohydrate found in cereals/pseudocereals was not detected in chia and was significantly lower in wholemeal flours of pseudocereals than in wheat flours (Table 2). This may be particularly relevant in cereal food formulations to lower their GI values [12].

### 3.2. Effect of the Independent Variables on Bread Nutritional Properties

When optimising a bread product formulation, the intention is to maximise lipid content because it is known that amaranth, quinoa, and chia flours have high polyunsaturated fatty acid (PUFA) contents with beneficial health effects, as previously reported [20,23]. The lipid content of all the studied formulations varied from 1% to 12% d.m. (dry matter) (Figure S1). This means that the isolated inclusion of chia flour in the formulation increased this parameter, just as its linear coefficient indicated (*a*_1_: 0.214, *p* < 0.01), followed by the quinoa (*a*_2_: 0.061, *p* < 0.01) and amaranth (*a*_3_: 0.002, *p* < 0.01) flours (Table 3). A significant interaction was observed between the amaranth and chia flours (*a*_13_: 0.002, *p* < 0.05), which was not detected with quinoa (Table 3). This effect could be shown in the baked products requiring high temperatures when baked, such as bread products containing amaranth and chia, as they present better lipid stability because both matrices contain high concentrations of antioxidants, unlike quinoa [20,39,40,43]. Natural antioxidants are present, such as tocopherol and squalene, although the latter is only present in amaranth [51] and can help to reduce lipid oxidation in chia [52]. Therefore, it would be worthwhile to mix chia and amaranth flours to protect the lipid fraction in formulations. Moreover, this interaction effect that took place in the chia–amaranth mixture, but not in the quinoa flour, could also be affected by quantities of polyunsaturated fatty acids (PUFAs), saturated fatty acids (SFAs), and ratios. As the PUFA/SFA ratio increases, lipid stability diminishes in oxidative rancidity terms [53]. The quinoa PUFA/SFA ratio was 4, while that of amaranth was 3 [40]. 

The calorie value is related mainly to lipid content. It rose as chia flour quantity increased, and, as expected, the linear coefficient was positive and significant (*a*_1_: 0.906; *p* < 0.01), followed by amaranth flour (*a*_3_: 0.307; *p* < 0.0), and finally by quinoa flour (*a*_3_: 0.129; *p* < 0.01). Protein and ash contents varied between 14.25–20.26% d.m. and 0.91–3.27% d.m., respectively. The nutritional value of formulations rose as the amount of chia, quinoa, and/or amaranth wholemeal flour did in formulations. This increase mostly responded to the effects of the linear coefficients in each studied ingredient *a*_1_ > *a*_2_ > *a*_3_, respectively (Table 3). However, the nutritional criterion was not the only one used to optimise bread product formulations because a direct relation appeared between the amount of each flour and the nutritional value. Moreover, the healthy fat content, proteins with higher biological values, and minerals were maximised, but the calorie value was minimised (Figure S2). Other quality criteria were also considered, such as the product’s technological and sensorial characteristics, in order to seek a compromise formulation that lived up to all expectations from a holistic viewpoint, as shown below. 

### 3.3. Effect of the Independent Variables on Bread Technological Characteristics

The specific volume of all the studied formulations varied from 0.90 ± 0.06 mL/g, which corresponded to the formulation with a higher substitution percentage (Ch_20_Q_40_A_40_), to 5.5 ± 0.1 mL/g (CB). This parameter was negatively affected when gluten-free ingredients were included and contained a higher proportion of fibre, which led to a poor retention of the carbon dioxide produced during fermentation [54,55]. As expected, the effect of linear coefficients indicated a smaller specific volume when the replacement rate rose for the amaranth (−0.087; *p* < 0.01), quinoa (−0.059; *p* < 0.01), and chia (−0.039; *p* < 0.01) flours (Figure S3). The highest specific volume value among formulations, after the control sample, was for the formulations with chia (Ch_10_: 5.0 ± 0.1 mL/g and Ch_20_: 4.9 ± 0.1 mL/g). Increasing chia substitution in wheat flour lowered gluten content, and the specific volume was also reduced, but not as significantly as in the formulations with amaranth or quinoa at the same substitution level (Am20%: 4.3 ± 0.1 and Q20%: 4.6 ± 0.1 mL/g, respectively). Moreover, the effect of gluten dilution and the difference in the specific volume among the formulations with chia, amaranth, and/or quinoa flours could have been due to the high proportion of mucilage that chia seeds possess, forming hydrophilic complexes among ion groups and gluten proteins [11]. This tendency has been observed in wheat bread with a 5% chia flour substitution whose specific volume was higher than for the chia-free control sample [11].

The crumb firmness parameter varied from 0.96 ± 0.03N (CB) to 29.59 ± 0.71 N (formulation with a higher degree of substitution, Ch_20_Q_40_A_40_). This parameter positively correlated with chia flour (0.381; *p* < 0.01), but with no interactions; that is, the more this ingredient was added, the higher crumb firmness values became (Ch_10_: 1.12 ± 0.06 N and Ch_20_: 9.0 ± 0.3 N). This result indicates that the inclusion of chia/mucilage does not always reduce/maintain crumb firmness, and it depends on the substitution level in formulations [11]. Firmness also increased with a bigger quantity of the quinoa (Q_40_: 2.29 ± 0.32 N) and amaranth (A_40_: 1.22 ± 0.41 N) wheatmeal flours, but to a lesser extent in the latter. Given the interaction between the inclusion of quinoa and amaranth flours, a synergy was generated with increased crumb firmness (Q_20_A_20_: 3.28 ± 0.13 N; Q_40_A_20_: 7.67 ± 0.43 N; Q_20_A_40_: 15.70 ± 0.08 N; Q_40_A_40_: 23.24 ± 0.42 N), as shown in Figure S4. A minimum value was obtained in this context according to the quadratic coefficient of the amaranth flour factor (*a*_33_: 0.008; *p* < 0.01), which would imply that the formulations substituted only for amaranth flour would have closer values to the control bread’s firmness values (0.96 ± 0.03 N). 

The shape ratio parameter (the width/height ratio of the central slice of a loaf) showed an interaction coefficient between the amaranth and chia flours (*a*_13_) with a reduction from 2.13 ± 0.02 cm/cm (CB) to 1.66 ± 0.03 cm/cm (Am_20_Ch_20_). Conversely, the interaction coefficient between the amaranth and quinoa flours (*a*_23_) left this parameter at 2.14 ± 0.28 cm/cm (Q_20_Am_40_); that is, loaves with the same shape, but a smaller volume. This would mean that formulations with amaranth and chia flours could be used to obtain more circular-shaped loaves. A similar tendency has been previously reported when substituting wheat for wholemeal amaranth flour [16]. An increase in the proportion of chia also affected the shape of the central slice, and a minimum was obtained according to the quadratic coefficients (0.001; *p* < 0.01) [11] (Figure S5).

**Figure 1:**
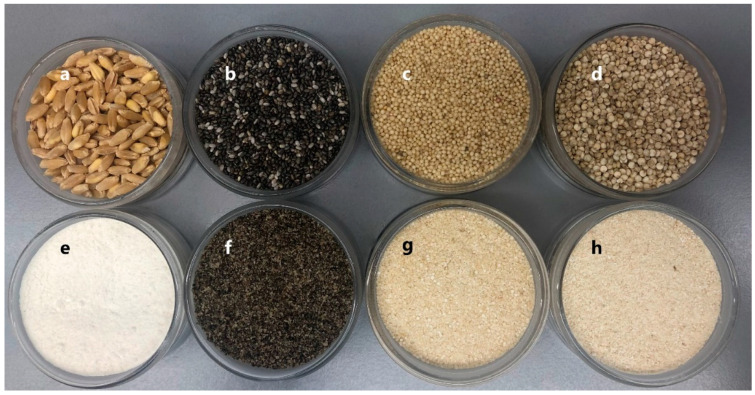
Raw materials: wheat grains (**a**); chia seeds (**b**); amaranth grains (**c**); quinoa grains (**d**); wheat flour (**e**); whole chia flour (**f**); whole amaranth flour (**g**); whole quinoa flour (**h**).

The difference in crust colour and crumb colour (ΔE*) varied within the 3.4 ± 0.8 to 25.0 ± 0.3 and 5.6 ± 0.3 to 20.2 ± 0.3 ranges, respectively, compared to the control sample. A loaf’s crust colour is given mostly by non-enzymatic browning, by the Maillard reaction, and by caramelisation to a lesser extent. Differences in crust colour among formulations were due mainly to raw materials’ colour, followed by different chemical reactions while baking that depend on the employed flour to a greater or lesser extent (Figure 1). Crumb showed fewer colour differences versus the control than crust because the speed of browning reactions slowed down due to higher humidity [56,57]. The linear coefficients of each ΔE* factor for crust and crumb were significantly relevant (Table 3). The most significant ingredient (*p* > 0.05) was chia flour, which can be easily explained by the raw materials’ colour parameter values (Figure 1). Chia flour displayed a more reddish (a*) and darker (lower L*) colouring than the other flours (chia: L* = 3.0 ± 1.3, a* = 2.2 ± 0.1, b* = 6.6 ± 0.1; quinoa: L* = 71.98 ± 0.07, a* = 1.42 ± 0.06, b* = 15.25 ± 0.01; amaranth: L* = 54.4 ± 2.1, a* = 2.13 ± 0.03, b* = 13.7 ± 0.4; wheat: L* = 65.8 ± 1.0, a* = −0.64 ± 0.04, b* = 7.5 ± 0.2) (Figure 1). The effect of the quinoa/amaranth interaction, which led to less marked crust colour differences, was lost when chia flour was added. Those formulations containing only amaranth and quinoa obtained crust ΔE* values of 11.5 ± 0.5, 12.1 ± 0.6, and 13.0 ± 1.0 (Q_20_A_40_, Q_40_A_20_, and Q_40_A_40_, respectively). However, this tendency was the opposite to that observed in flours, and the highest colour change value was found for amaranth flour (ΔE* = 14.7 ± 0.3) instead of for quinoa flour (ΔE* = 9.66 ± 0.06) when both were compared to wheat flour. The biggest colour difference for amaranth flour can be explained by the red-violet pigment of the betacyanins present in *Amarantus caudatus*, which confers its grain a slightly redder colour, unlike white quinoa in which this pigment was not detected [40,58,59]. Therefore, the less marked colour change in the formulations with amaranth flour could be because this pigment inhibits the Maillard reaction and avoids final glycosylation products from being produced [60]. Finally, the formulations substituted for up to 40% quinoa flour reached a maximum with chia in the Q_40_Ch_20_ formulation (ΔE* = 22.4 ± 0.4), as the quadratic coefficient indicated.

### 3.4. Preliminary Sensorial Evaluation

The scores in all the studied formulations for all the investigated sensorial attributes were similar to or lower than those obtained by the control bread, but were never superior. The linear coefficients of the sensorial attributes studied for the quinoa factor indicated that the more this ingredient was added, the worse the loaf, texture, flavour, and the product’s overall acceptability became (Table 3). However, when amaranth was included, consumers gave crumb texture and the product’s overall acceptability acceptable values. According to the 9-point hedonic scale, the texture attribute scores varied from 3.40 ± 0.05 to 8.5 ± 0.7, and the product’s overall acceptability score went from 2.7 ± 0.5 to 8.4 ± 0.2. The higher scores of these two attributes were for the control bread, followed by formulation A_20_ (texture: 6.3 ± 0.2 and overall acceptance: 7.3 ± 0.3 between “Moderately like” and “Very much like”), which remained some way behind those obtained in control bred (CB) (texture: 8.5 ± 0.7 and overall acceptance: 8.4 ± 0.2, between “Very much like” and “Especially like”) (Table 3). No significant interaction was detected between either quinoa and chia (a_12_) or quinoa and amaranth (a_23_).

According to the linear coefficient (*a*_1_), as the proportion of chia flour increased in the formulation, bread appearance slightly improved, with a maximum in the experimental domain (Ch_10_A_20_: 7.67 ± 0.08) in accordance with the quadratic coefficient (*a*_13_), but it had no effect on texture, flavour, and acceptability. Conversely, for higher quinoa substitutions, sensorial parameters were negatively influenced (Figure S6).

### 3.5. Optimising Bread Formulation

To obtain the optimum formulation, we assigned conditions per response: (1) for the nutritional value, the lipid, protein, and ash contents were maximised, and the product’s calorie value was minimised; (2) for technological quality, the loaf-specific volume was maximised, while the central slice aspect/crumb firmness ratio, as well as differences in the crust colour and crumb colour parameters compared to the control sample, were minimised; (3) consumer scores given to the sensorial attributes appearance, texture, flavour, and the baked product’s overall acceptability were maximised. The formulation with all these attributes was formulation Ch_10_Q_4_A_19_ (chia 10%, quinoa 4%, amaranth 19%), that is, a loaf made with wheat flour substituted for 33% wholemeal chia, quinoa, and amaranth flours at the 10%, 4%, and 19% flour basis proportions, respectively. 

To validate the model, the optimum formulation was experimentally obtained and indicated Ch_10_Q_4_A_19_. Its nutritional, technological, and sensorial characteristics were compared to the values that the model predicted. Hence, the obtained experimental results differed from those predicted by a percentage error between 0.52% and 6.7%, except for the two sensorial attributes (Table 4). A comparative study was also conducted between the characteristics of the optimised formulation and the bread made with the refined wheat and wholemeal wheat flours (Table 4). 

#### 3.5.1. Evaluating the Quality Parameters of the Optimised Bread

A comparison of the physico-chemical and sensorial characteristics of the wholegrain wheat bread, refined wheat (the control sample), and optimised bread can be found in Table 4. The addition of quinoa, chia, and/or amaranth flours significantly increased soluble, insoluble, and total fibre compared to those found in the wholemeal and control breads. In the latter, the difference was obvious because refined flours lack both the germ fraction and outer cereal layers. Unrefined flours are rich in dietary fibres, lipids, and minerals, and are complete in nutrients and beneficial bioactive compounds that benefit our health [61]. As expected, the optimised formulation indicated similar protein and lipid contents to the wholemeal bread, but higher ones than in the control bread. The optimised bread’s calorie value (259 ± 4 Kcal/100 g) was lower than that for the wholemeal bread (279 ± 1.2 Kcal/100 g) despite similar lipid contents. This was due to the high fibre content in the optimised formulation (15.0 ± 1.3% d.m) compared to the wholemeal wheat bread (7.0 ± 0.5% d.m), and also due to its lower carbohydrate content, which would imply a considerable reduction in its calorie value. This means that the optimised bread could be considered fibre-rich food with a similar common bread value despite the high degree of substitution for flours with high lipid proportions. 

Regarding crumb texture, the optimised formula generally had a similar profile to that of the wholemeal wheat bread. Although elasticity and chewiness presented no significant differences among formulations, a lowering trend was observed in both parameters compared to the control bread. The crumb firmness parameter differed significantly among formulations, where the optimised bread formulation obtained an intermediate value between the control and wholemeal wheat breads (Table 4). 

**Figure 2:**
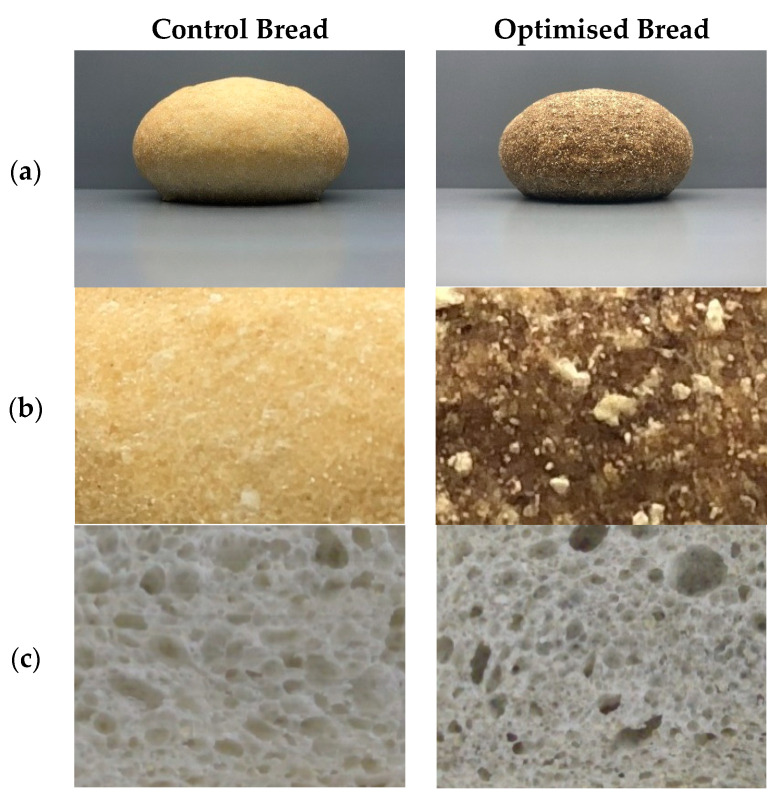
Photographic images of control bread and optimised bread: (**a**) bread roll, (**b**) crust, (**c**) crumb structure.

For crumb structure, the mean crumb cell area in the wholemeal wheat bread was significantly higher than the values obtained for the control and optimised breads (Table 4). This tendency was also found by Angioloni and Collar [62] when they compared crumb cell distributions in the bread made with wholemeal and refined wheat flours. No significant differences appeared among the formulations for the cell area/total area parameter (Table 4). Nonetheless, in the optimised bread, this parameter was slightly lower with the subsequent significant increase in the wall area/total area compared to the control. This result might be related to the 33% gluten dilution in the optimised formulation, which negatively interfered with CO_2_ retention capacity during bread-making fermentation and oven impulse, which leads to more compact crumbs and a smaller specific volume in the optimised product versus the control (Figure 2). 

The difference in the crumb/crust colour (ΔE*) of the optimised and wholemeal wheat breads, in comparison with the control bread, exceeded a value of 5. This indicates that consumers would notice colour differences at a glance (Table 4). The results of colour parameters L*, C*, and *h_ab_* for the wholemeal bread crust were similar to those for the optimised bread (L* and *h_ab_*), but significantly differed from those of the control bread. The optimised bread crust was a darker colour, conferred mostly by the chia flour included in the formulation (Figure 2). A similar tendency was found when replacing 5% wholemeal flour with wholemeal chia flour [11]. Nevertheless, the crumb luminosity in the optimised formulation was lower than for the wheat bread, with similar saturation to the control bread, along with an intermediate tone somewhere between the control formulation and wholemeal wheat. 

Overall consumer acceptability showed no significant differences among formulations, with scores ranging from 8.1 ± 0.9 (wholemeal bread) to 8.7 ± 0.8 (optimised formulation) between “Very much like” and “Especially like”.

#### 3.5.2. Nutritional Properties of the Optimised Bread

##### Contribution of Bread Minerals to Diet

The nutritional quality of wholemeal flours with these ancient seeds is generally higher than those of cereals, not only for their high mineral content but also for their content of dietary fibre, unsaturated fatty acids, and proteins of high biological value [12]. Nonetheless, the high concentration of some antinutrients, including phytic acid or phytates (Ins*P*_6_), affects the bioavailability of the minerals in the human intestine, which is indicated by the inhibition threshold values given for the Ins*P*_6_/mineral molar ratio [63]. Phytate inhibition is basically due to insoluble complexes forming in the digestive tract, and solubility is a major requirement when these complexes are absorbed by enterocytes in the intestine [64]. Nonetheless, phytate content gradually decreases as the bread-making process advances owing to endogenous phytase activity. This activity depends on a number of factors, including fermentation time and temperature, dough pH, and baking time. Yet, the reduction of phytates via endogenous phytases during the bread-making process is not generally enough to extensively improve the bioavailability of minerals [65]. Thus, in order to estimate the contribution that each mineral makes to adequate intake (AI) in our daily diet when we eat bread products it is necessary to quantify this antinutritional compound, as well as its possible inhibition in the bioavailability of minerals by focusing on Zn. 

Eating 100 g of bread made with the optimised formula on a daily basis can contribute to suitable Ca intake, which was threefold higher than in the control bread, by being completely bioavailable and having an Ins*P*_6_/Ca ratio below 0.24 (Table 5). With regards to Zn content, according to the FAO/WHO [66], its contribution to diet is contemplated in accordance with a high, moderate, or low degree of bioavailability, which depends on the Ins*P*_6_/Zn < 5, 5 < Ins*P*_6_/Zn < 15, or Ins*P*_6_/Zn > 15 molar ratio, respectively. The Zn content of the optimised formulation and the control bread displayed high bioavailability, and the former stood out for contributions of 29% in men and 41% in women (Table 5). Other bread formulations with 10% chia wholemeal flour or 25% wheatmeal quinoa flour substitutions have presented a moderate/high bioavailability with high contributions, but their contribution was not as high as that reported herein [12,14]. 

With regards to Fe contribution, it can be stated that Fe bioavailability in both formulations is compromised. Studies about Fe bioavailability in vitro using the Caco-2 cell line model were studied. They revealed Fe bioavailability inhibition in bread to which wholemeal amaranth flour had been added up to the 40% substitution level [18]. Nonetheless, 20% substitution with wholemeal amaranth flour led to better Fe absorption compared to the control bread; that is, although mineral absorption was inhibited, the higher Fe content favoured its higher bioavailability in the study model [18]. Thus, a higher proportion of Fe in the optimised formulation could contribute to increasing Fe bioavailability owing to other involved mechanisms apart from the inhibition caused by phytates. Low Fe bioavailability has been previously observed in the formulations of bread with Ins*P*_6_/Fe ratios above 1 and the following replacements: 25% wholemeal quinoa flour, 10% chia flour, 30% amaranth [12,14,21].

##### Fatty Acid Quality of Bread

The optimised bread with a higher lipid proportion stood out for its high PUFA content compared to the control bread. The PUFA/SFA ratio went from 0.66 in the control bread to 5.8 in the optimised formulation. Therefore, given its bigger PUFA supply, it can be considered a healthy product [67]. The majority PUFA content in the optimised bread corresponded to the alpha-linolenic acid content (C18:3n3; 1.5%), which was higher in the wheat bread with the 10% chia flour substitution (C18:3n3; 0.13%) [12]. What this shows is that the chia, amaranth, and quinoa flours mixture enhances the PUFA/SFA ratio, even after the changes that the bread-making process entails. This means that such functional products could positively impact health after eating these flours because of the lower low-density lipoprotein (LDL) cholesterol concentration and due to the total cholesterol/high-density lipoprotein (HDL) cholesterol ratio, which reduce the risk of developing cardiovascular diseases [67,68]. 

Fatty acids omega-6 and omega-3 can be synthesised by means of their precursors alpha-linolenic acid (ALA) and linoleic acid (LA), respectively [20]. For adults, the daily intakes of 10 g of LA/day and 2 g of ALA/day have been established [71]. As far as nutrition and preventing cardiovascular diseases are concerned, food agencies recommend maintaining the LA/ALA ratio in diet between 1:1 and 5:1 [72]. The LA/ALA ratio of 0.6:1 obtained with the optimised formula was even better than these recommendations and the control bread ratio (LA/ALA, 17:1). Other formulations obtained higher ratios than the recommended ratio, e.g., those with 25% wholemeal quinoa flour of different varieties (LA/ALA, 9.2:1-9.6:1), and were better than in the wheat formulations [14]. The formulations with 10% wholemeal chia flour obtained ratios that agree with recommendations (LA/ALA; 2.4:1), and a similar tendency has been reported for bread made with 15% salmon powder (LA/ALA, 3.4:1) [12,73]. This indicates that a mixture with suitable levels of wholemeal flours from pseudocereals and oilseeds in a bread product formulation would improve this ratio, as we observed with our optimised bread. Moreover, a daily intake of 100 g of optimised bread would cover the recommended alpha-linolenic acid intake (ALA, >0.5%E) and supply 9% of the recommended linoleic acid intake (LA, 2.5%E) (Table 5). The daily intake of the optimised bread product could prevent coronary heart diseases and metabolic disorders [67].

##### In Vitro Protein Digestibility (IVPD), Protein Digestibility Corrected Amino Acid Score (PDCAAS), and Nutritional Index of Bread

The IVPD of the optimised bread was lower than in the control bread (Table 5), which could be explained by the presence of antinutrients such as phytic acid that form complexes with digestive proteins by lowering their digestibility and bioavailability for the metabolism, despite the content of this compound being lowered during the bread-making process. It is worth pointing out that the protein digestibility of the optimum formulation (IVPD, 73%) was similar to the true digestibility of ready-to-eat cereals, based on corn, wheat, rice, or oat (IVPD, 70–77%), as reported by Gilani et al. [74]. The protein quality of the optimised bread was better than that of wheat bread, as indicated by the protein digestibility-corrected amino acid score (PDCAAS) used by FAO/WHO/United Nations University (UNU) [35]. Although the essential amino acids index (EAAI) of the optimised bread showed a significant difference with respect to the control bread, it tended to rise, indicating a protein of greater nutritional quality. Of the indices used to evaluate the nutritional value of foods, we found the nutritional index (NI), which combines qualitative and quantitative factors. It is considered an overall predictor of protein quality, and it was significantly (*p* < 0.05) higher for the optimised bread than the control bread. This same tendency has been previously reported when adding quinoa flour to wheat bread [75].

##### Dietary Fibre Content in Bread

Generally, pseudocereals such as quinoa and amaranth, and oilseeds such as chia, are an excellent source of dietary fibre [15,42,76,77]. The optimised formula obtained 54% and 64% more total fibre compared to the wholemeal and the control wheat bread, respectively (Table 4). The total dietary fibre of our optimised formulation (15.0 ± 1.3% d.m.) was higher than that of the wholemeal flour formulations, to which sugar beet pulp and apple marc were added to increase the fibre content in the end product without making product acceptability worse [78]. The higher proportion of soluble fibre in the optimised formulation compared to the wholemeal wheat bread could be due to the addition of amaranth, quinoa, and chia with a higher proportion of soluble fibre, as described in the literature and as obtained in the present study [77,79]. Chia in particular contains a higher proportion of soluble fibre in the form of mucilage, which can absorb up to 35.2-fold its weight in water [77]. 

The physiological action that occurs after eating food containing dietary fibre is more effective when the soluble/insoluble fibre ratio remains at 1:2 [70]. This ratio of the optimised formulation came close to the recommended one, unlike the control bread (Table 5). Thus, eating 100 g/day of optimised bread would contribute up to 60% of the total suitable dietary fibre intake for adults (25 g/day) [69]. The combined use of wholemeal quinoa/amaranth/chia flours as ingredients to prepare bread products is an important source of balanced dietary fibre to cover, to a great extent, the daily recommended fibre intake, given its beneficial effects for the organism, such as lowering cholesterol, reducing the risk of developing diabetes, and regulating intestinal passage [70].

##### In Vitro Starch Digestion Analysis

The optimised bread’s starch content was significantly lower than in the control bread (Table 5). This could partly explain why after 90 min after eating starch in wheat bread, 84.6% of hydrolysed starch was recorded, but only 68.1% of hydrolysed starch was found in the formulation with the chia/quinoa/amaranth flours (optimised bread) (Table 5). These differences in starch hydrolysis speed did not only respond to the initial starch content, but also to granule type and size, the degree of gelatinisation, and other components present in the food matrix, such as fibre, proteins, and lipids, which wholemeal flours normally contain [80]. All these factors influence the GI in foods to a greater or lesser extent. The control bread had a 95% GI, which was 85% for the optimised formulation (Table 5). These values are similar to the GI values of the buckwheat (GI = 80) or quinoa (GI = 95) formulations [38]. A lower GI can be explained by the higher soluble fibre content in the optimised bread compared to the control bread (Table 4), and a similar tendency has been observed in biscuits enriched with soluble fibre, which obtained a lower GI than their non-soluble fibre counterpart [81]. The authors suggested that the effectiveness of soluble fibre in regulating the postprandial glycaemia response was attributed to its capacity to delay gastric emptying. This tendency has also been observed in gluten-free bread products where, apart from replacing refined flour and starch, viscous diet fibres reduced the glycaemic response thanks to the capacity to form hydrogen links with water [82]. Laparra and Haros [80] confirmed that the inclusion of amaranth, quinoa, and chia flours in bread can significantly lower GI values. The reason for this could be the presence of antioxidants in amaranth and quinoa flours, activating the metabolism of carbohydrates and preventing diseases such as type 2 diabetes, obesity, and hypertension [45,83].

### 3.6. Conclusions

The response surface model herein employed was suitable for optimising bread formulations from the nutritional, technological, and sensorial points of view. Combining healthy ingredients such as chia, quinoa, and amaranth helped to develop a product with optimum characteristics (Ch_10_Q_4_A_19_: chia 10%, quinoa 4%, amaranth 19%) as far as its nutritional characteristics were concerned (higher PUFA/SFA ratio, higher dietary fibre content by supplying soluble fibre, larger mineral supply, lower GI and better NI). It is a product with acceptable and better quality parameters than wholemeal wheat products (in terms of crumb firmness, loaf-specific volume, and the shape ratio of the central slice), and with excellent sensorial characteristics that are similar to products made with refined wheat flour. This optimised product can contribute to providing products with a beneficial impact on consumer health with high acceptability.

## Figures and Tables

**Table 1:** Factorial design.

Trial	Name	% of Substitution in Flour Basis			Variables Codes		
		Chia Flour	Quinoa Flour	Amaranth Flour	Chia *x*_1_	Quinoa *x*_2_	Amaranth *x*_3_
1	CB	0	0	0	−1	−1	−1
2	Ch_10_	10	0	0	0	−1	−1
3	Ch_20_	20	0	0	1	−1	−1
4	Q_20_	0	20	0	−1	0	−1
5	Ch_10_Q_20_	10	20	0	0	0	−1
6	Ch_20_Q_20_	20	20	0	1	0	−1
7	Q_40_	0	40	0	−1	1	−1
8	Ch_10_Q_40_	10	40	0	0	1	−1
9	Ch_20_Q_40_	20	40	0	1	1	−1
10	A_20_	0	0	20	−1	−1	0
11	Ch_10_A_20_	10	0	20	0	−1	0
12	Ch_20_A_20_	20	0	20	1	−1	0
13	Q_20_A_20_	0	20	20	−1	0	0
14	Q_20_A_20_Ch_10_	10	20	20	0	0	0
15	Q_20_A_20_Ch_20_	20	20	20	1	0	0
16	Q_40_A_20_	0	40	20	−1	1	0
17	Q_40_A_20_Ch_10_	10	40	20	0	1	0
18	Q_40_A_20_Ch_20_	20	40	20	1	1	0
19	A_40_	0	0	40	−1	−1	1
20	A_40_Ch_10_	10	0	40	0	−1	1
21	A_40_Ch_20_	20	0	40	1	−1	1
22	Q_20_A_40_	0	20	40	−1	0	1
23	Q_20_A_40_Ch_10_	10	20	40	0	0	1
24	Q_20_A_40_Ch_20_	20	20	40	1	0	1
25	Q_40_A_40_	0	40	40	−1	1	1
26	Q_40_A_40_Ch_10_	10	40	40	0	1	1
27	Q_40_A_40_Ch_20_	20	40	40	1	1	1

CB: control bread; Ch: whole chia flour; Q: whole quinoa flour; A: whole amaranth flour.

**Table 2:** Chemical composition of raw materials.

Parameters ^a^	Units	Whole Wheat Flour	Wheat Flour	Whole Amaranth Flour	Whole Quinoa Flour	Whole Chia Flour
Moisture	%	13.25 ± 0.011 e	11.38 ± 0.09 b	12.41 ± 0.04 c	12.62 ± 0.04 d	7.95 ± 0.01 a
Protein	% d.m.	11.76 ± 0.07 a	12.54 ± 0.1 b	17.02 ± 0.10 c	17.51 ± 0.50 c	19.63 ± 0.3 d
Lipids	% d.m.	1.45 ± 0.03 b	1.00 ± 0.02 a	6.60 ± 0.20 d	6.45 ± 0.02 c	34.2 ± 0.4 e
Ash	% d.m.	1.43 ± 0.02 b	0.58 ± 0.01 a	2.65 ± 0.04 d	2.82 ± 0.05 c	4.69 ± 0.05 e
Starch	% d.m.	73.3 ± 3.00 b	68.9 ± 2.90 b	55.0 ± 0.30 a	54.30 ± 1.70 a	ND
Total fibre	% d.m.	6.58 ± 0.06 b	3.90 ± 0.10 a	15.6 ± 2.90 c	14.20 ± 0.60 c	39.0 ± 0.1 d
Soluble dietary fibre	% d.m.	0.88 ± 0.07 a	1.06 ± 0.46 b	3.08 ± 1.50 b	4.10 ± 1.20 b	6.60 ± 1.30 c
Insoluble dietary fibre	% d.m.	5.70 ± 0.05 b	2.81 ± 0.35 a	12.6 ± 1.4 d	10.2 ± 0.5 c	32.4 ± 1.3 e

^a^ Mean ± SD *n* = 3. Values followed by the same letter in the same column are not statistically different at 95% confidence level; d.m., dry matter; ND, not detected.

**Table 3:** Factorial design coefficients of nutritional, technological, and sensory properties of bread.

	Physicochemical Properties				Technological Properties					Sensory Evaluation			
Source	Lipids	Ash	Protein	Caloric Value	Specific Volume	Crumb Colour, ∆E *	Crust Colour, ∆E *	Crumb Firmness	Shape Ratio	Appearance	Texture	Taste	Overall Acceptability
Units	% d.m.	% d.m.	% d.m.	kcal/100 g	mL/g	—	—	N	cm/cm	—	—	—	—
*a*_0_	−0.227	0.900	14.877	250.345	5.691	2.100	0,345	1.287	2.144	7.711	7.423	8.090	8.008
*a*_1_	0.214 **	0.041 **	0.070 **	0.906 **	−0.039 **	0.721 **	0.815 **	0.381 **	−0.037 **	0.005 **			
*a*_2_	0.061 **	0.035 **	0.047 **	0.129 **	−0.059 **	0.349 **	0.656 **	−0.129 **	−0.014 **	−0.051 **	−0.0001 **	−0.053 **	−0.055 **
*a*_3_	0.059 **	0.027 **	0.048 **	0.307 **	−0.087 **	0.144 **	0.165 *	−0.254 **			0.037 **		0.042 **
*a*_11_	0.005 *			0.033 **					0.001 *	−0.009 *			
*a*_12_						−0.007 **	−0.006 *						
*a*_13_	0.002 *	0.001 *							−0.001 **	0.003 *	0.003 **	0.003 *	0.004 **
*a*_22_							−0.007 **		0.001 **				
*a*_23_					0.001 *		−0.006 **	0.011 **	−0.0003 **				
*a*_33_		−0.0004 **						0.008 **			−0.002 **	−0.003 **	−0.001 **
_R-SQ_	0.985	0.967	0.979	0.982	0.931	0.933	0.925	0.931	0.936	0.789	0.789	0.653	0.703

Codes: 0.05 (*) and 0.01 (**) indicate statistical significance at the 95 and 99% confidence levels, respectively. d.m., dry matter, “—” without units. ∆E*: total colour difference, *a*_1_, *a*_2_, and *a*_3_ are the coefficients of the main single effects of *x*_1_, *x*_2_, and *x*_3_, respectively (*x*_1_ is whole chia flour, *x*_2_ is whole quinoa flour, and *x*_3_ is whole amaranth flour). The square coefficients (*a_ii_*) indicate if any of the variables has a maximum or minimum in the experimental domain, whereas the mixed coefficients (*a_ij_*) represent the interactions between factors. R-SQ: adjusted square coefficient of the fitting model.

**Table 4:** Physico-chemical and sensory characteristics of bread formulations.

Parameters	Units	Bread Formulation				
		Whole Wheat	Control	Optimal Formulation	Predicted Value	*Δ*%
Physico-chemical parameters ^a^						
Lipids	% d.m.	3.6 ± 0.6 b	0.23 ± 0.03 a	3.4 ± 0.1 b	3.500	−2.941
Ash	% d.m.	2.3 ± 0.5 b	0.91 ± 0.10 a	2.6 ± 0.1 b	2.500	3.846
Protein	% d.m.	12.3 ± 1.6 a	14.3 ± 0.04 ab	15.8 ± 0.1 b	16.400	−3.145
Soluble dietary fibre	% d.m.	1.6 ± 0.1 a	1.07 ± 0.04 a	4.1 ± 0.7 b	n.i.	--
Insoluble dietary fibre	% d.m.	5.4 ± 0.4 a	4.4 ± 0.3 a	10.9 ± 0.6 b	n.i.	--
Total dietary fibre	% d.m.	7.0 ± 0.5 a	5.4 ± 0.4 a	15.0 ± 1.3 b	n.i.	--
Caloric values	Kcal/100 g	279 ± 1.2 b	250 ± 4 a	259 ± 4 a	268	−3.520
Ins*P*_6_	mg/100 g	10.7 ± 1.8 b	1.5 ± 0.6 a	3.4 ± 0.4 a	n.i.	--
Technological parameters ^a^						
Specific volume	mL/g	3.03 ± 0.03 a	4.5 ± 0.3 b	3.6 ± 0.4 ab	3.690	−2500
Shape ratio	cm/cm	1.6 ± 0.1 a	2.13 ± 0.02 a	1.7 ± 0.2 a	1.840	−8.235
Crumb textural parameters (TPA) ^a^						
Firmness	N	2.26 ± 0.02 c	0.96 ± 0.03 a	1.95 ± 0.03 b	1.991	2.513
Springiness	mm	1.00 ± 0.00 a	1.70 ± 0.11 a	1.01 ± 0.26 a	1.000	1.478
Cohesiveness	m/m	0.79 ± 0.00 ab	0.84 ± 0.00 b	0.73 ± 0.03 a	0.750	−2.740
Chewiness	N	1.90 ± 0.01 a	1.46 ± 0.61 a	2.06 ± 0.96 a	2.020	1942
Crust colour parameters ^b^						
L*	—	49.7 ± 1.0 a	61.2 ± 2.0 b	51.7 ± 1.9 a	51.390	0.523
C*	—	31.99 ± 0.02 b	34.4 ± 1.08 c	27.4 ± 0.4 a	28.860	−5.175
*h_ab_*	—	60.7 ± 0.5 a	74.0 ± 2.0 b	71.8± 0.4 a	68.870	4.081
∆E*	—	17.9 ± 0.2 b	—	10.5 ± 0.5 a	10.030	4.111
*Crumb colour parameters ^b^*						
L*	—	57.6 ± 1.3 b	61.5 ± 1.2 b	52.6 ± 0.1 a	52.140	0.875
C*	—	20.7 ± 0.9 b	13.3 ± 0.8 a	15.0 ± 0.2 a	15.280	−1.867
*h_ab_*	—	78.5 ± 1.2 a	95.1 ± 0.8 c	85.9 ± 0.1 b	86.670	−0.896
∆E*	—	18.5 ± 2.1 b	—	9.35 ± 0.08 a	9.980	−6.738
*Crumb structure ^a^*						
Cell area/total area	cm^2^/cm^2^	0.40 ± 0.10 a	0.24 ± 0.00 a	0.21 ± 0.00 a	n.i.	--
Wall area/total area	cm^2^/cm^2^	0.60 ± 0.00 a	0.76 ± 0.00 b	0.79 ± 0.00 c	n.i.	--
Cells/cm^2^	—	402 ± 6 b	85 ± 7 a	109 ± 6 a	n.i.	--
Mean cell area	mm^2^	0.41 ± 0.02 c	0.28 ± 0.02 b	0.19 ± 0.01 a	n.i.	--
*Sensory analysis (hedonic scale) ^c^*						
Aspect	—	n.d.	8.5 ± 0.7 a	8.8 ± 0.8 a	8.540	2.955
Texture	—	n.d.	8.5 ± 0.7 a	8.5 ± 0.8 a	7.100	16.471
Taste	—	n.d.	8.7 ± 0.5 a	7.9 ± 0.7 a	7.460	5.570
Overall acceptability	—	8.1 ± 0.9 a	8.4 ± 0.2 a	8.7 ± 0.8 a	7.170	17.586

d.m.: dry matter; n.d.: not determined; n.i.: not included in the optimisation; Ins*P*_6_: *myo*-inositol hexakisphosphate or phytic acid; N: newton; L*: lightness; C*: chroma; *h_ab_*: hue angle; ∆E*: total colour difference, ∆E* = [(*∆L*)^2^ + (∆a*)^2^ + (∆b*)^2^]^1/2^; a*: redness to greenness; b*: yellowness to blueness; “—” without units. Mean ± SD, ^a^ *n* = 3, ^b^ *n* = 4, ^c^ *n* = 50. Values followed by the same letter in the same column are not statistically different at 95% confidence level.

**Table 5:** Nutritional composition of control and optimised breads.

Parameters		Reference Values (Male/Female)	Units	Control Bread	Optimised Bread
Average requirement (AR) contribution of minerals ^a^					
Ca		1000 mg/d	%	3	9
Fe		14/29 mg/d	%	5/2.4	12/6
Zn	High bioavailability	4.2/3 mg/d	%	14/20	29/41
	Moderate bioavailability	7/4.9 mg/d	%	9/12	18/25
	Low bioavailability	14/9.8 mg/d	%	4/6	9/13
Ins*P*_6_/Ca		<0.24	mol/mol	0.061	0.041
Ins*P*_6_/Fe		<1.0	mol/mol	2.123	2.014
Ins*P*_6_/Zn		<15.0	mol/mol	2.453	2.742
Fatty acids quality ^b^					
PUFA/SFA ratio				0.661	5.802
% of contribution of AI_LA_ E% for linoleic acid		2.5–9%E	%	5.998	9.210
% of contribution of AI_ALA_ E% for α-linolenic acid		>0.5%E	%	7.009	116,000
Ratio ω-6/ω-3		5:1		17:1	0.6:1
Protein quality ^c^					
In vitro protein digestibility (IVPD)			%	77.1 ± 0.3 b	72.8 ± 0.7 a
Protein digestibility corrected amino acid score (PDCAAS)				0.15 ± 0.01 a	0.44 ± 0.01 b
Essential amino acid index (EAAI)				2.4 ± 0.14 a	2.73 ± 0.09 b
Nutritional index (NI)				0.34 ± 0.02 a	0.43 ± 0.02 b
Adequate intake of dietary fibre ^d^					
Soluble/insoluble dietary fibre ratio		1:2	g/g	1:4.1	1:2.7
Adequate intake (AI_F_) contribution		25 g/d	%	21.673	60.026
In vitro starch digestibility ^e^					
Starch			% d.m.	66.5 ± 0.7 b	63.3 ± 0.1 a
TSH_90_: total starch hydrolysed at 90 min			%	84.6 ± 0.1 b	68.1 ± 2.0 a
AUC: area under the curve of starch digestion				5934 ± 83 b	4903 ± 81 a
GI: glycaemic index				95.0 ± 0.8 b	85.0 ± 0.8 a
pGL: predicted glycaemic load			%	28.3 ± 1.2 b	21.9 ± 0.6 a

^a–e^ Values are expressed as mean ± standard deviation (*n* = 3). Values followed by the same letter in the same line are not significantly different at 95% confidence level. ^a^ AR (average requirement) contribution (%) for a daily average intake of 100 g of bread. AR in milligrams per day for males/females ≥18. ^b^ PUFA: total polyunsaturated fatty acids, SFA: total saturated fatty acids AI (adequate intake) contribution (%) for a daily average intake of 100 g of bread. AI_LA or ALA_ E% (percentage of energy intake) for LA (linoleic acid) or ALA (α-linolenic acid) for adult ≥ 18, respectively, E = (Kcal proteins + Kcal lipids + Kcal carbohydrates) in 100 g of bread [69]. ^c^ PDCAAS: ASS (lowest score of an individual amino acid) x in vitro protein digestibility of bread sample; amino acid pattern suggested by Food and Agriculture Organisation (FAO) for adults (g/100 g protein). ^d^ Soluble/insoluble dietary fibre ratio, 1:2 g/g [70], AI_F_ (adequate intake) contribution (%) for a daily average intake of 100 g of bread. AI_F_ in adult ≥18 is 25 g/d [69]. ^e^ GI high > 70, medium 55–70, low < 55.

## Supplementary Materials

The following are available online at https://www.mdpi.com/2304-8158/9/12/1859/s1: Figure S1. Influence of interaction between the factors chia flour $\left(X_{1}\right)$, quinoa flour $\left(X_{2}\right)$, and amaranth flour $\left(X_{3}\right)$ on bread lipid yield. Figure S2. Influence of interaction between the factors chia flour $\left(X_{1}\right)$, quinoa flour $\left(X_{2}\right)$, and amaranth flour $\left(X_{3}\right)$ on bread calorie value. Figure S3. Influence of interaction between the factors chia flour $\left(X_{1}\right)$, quinoa flour $\left(X_{2}\right)$, and amaranth flour $\left(X_{3}\right)$ on bread specific volume. Figure S4. Influence of interaction between the factors chia flour $\left(X_{1}\right)$, quinoa flour $\left(X_{2}\right)$, and amaranth flour $\left(X_{3}\right)$ on crumb firmness. Figure S5. Influence of interaction between the factors chia flour $\left(X_{1}\right)$, quinoa flour $\left(X_{2}\right)$, and amaranth flour $\left(X_{3}\right)$ on shape ratio. Figure S6. Influence of interaction between the factors chia flour $\left(X_{1}\right)$, quinoa flour $\left(X_{2}\right)$, and amaranth flour $\left(X_{3}\right)$ on sensorial evaluation aspect.
