# Supplementary material for: Combined Effect of Chia, Quinoa and Amaranth Incorporation on the Physico-Chemical, Nutritional and Functional Quality of Fresh Bread
Source: Foods. 2020 Dec 12;9(12):1859. doi: 10.3390/foods9121859 (PMC7764627; doi:10.3390/foods9121859)
Supplement: Supplementary file 1 [file foods-09-01859-s001.pdf]

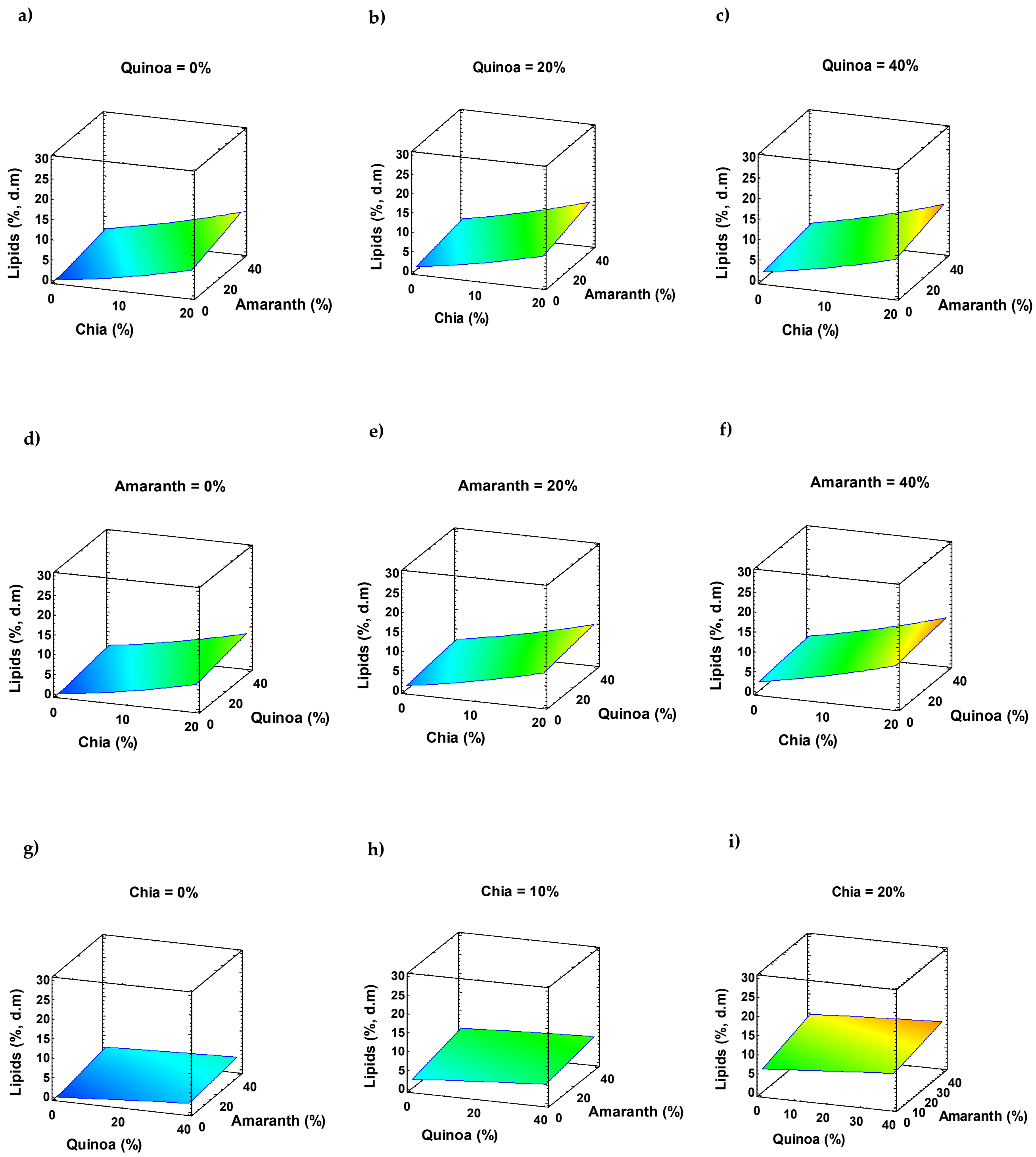

Figure S1. Influence of interaction between the factors Chia flour (X_1), Quinoa flour (X_2) and Amaranth flour (X_3) on bread lipid yield

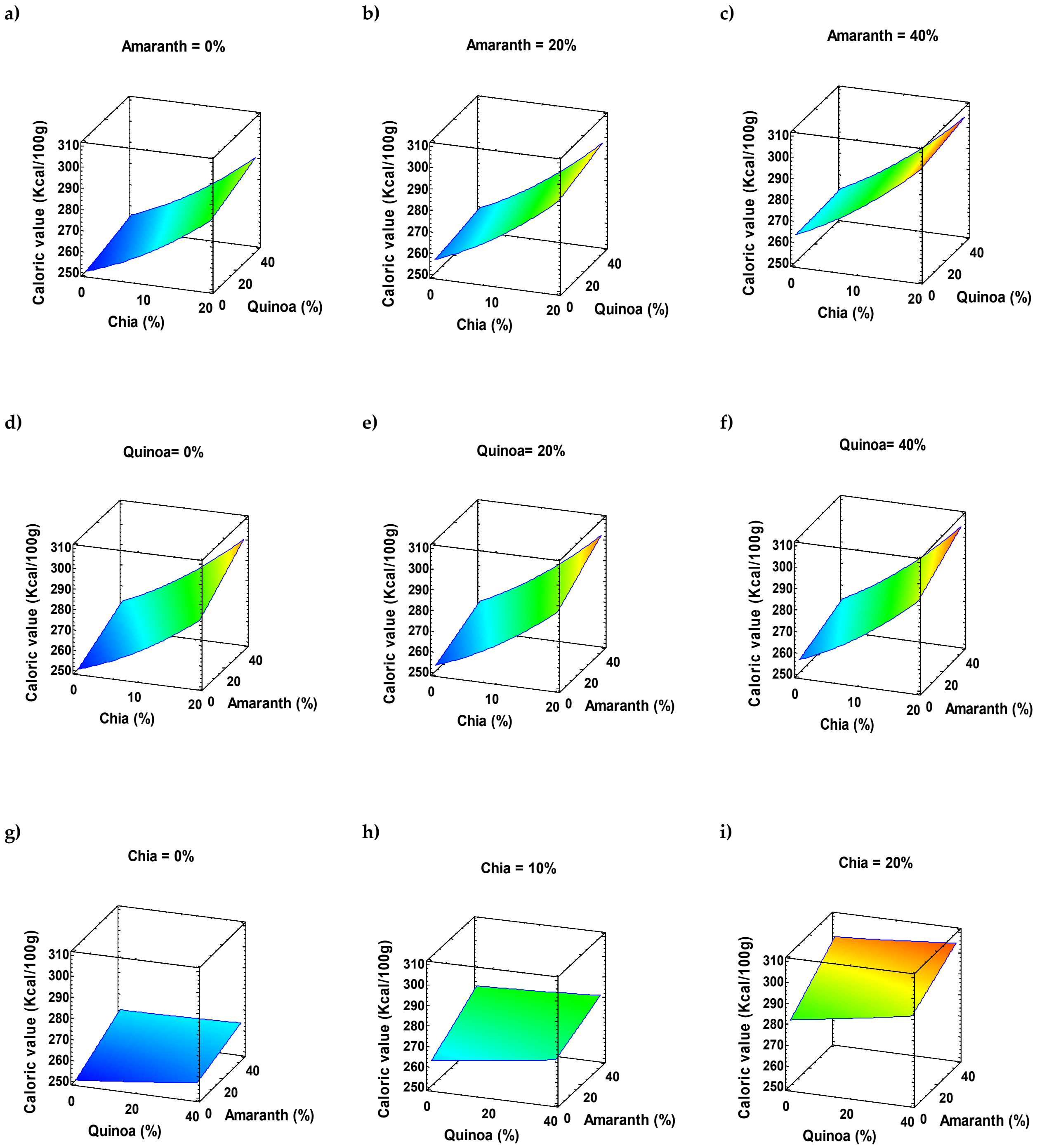

Figure S2. Influence of interaction between the factors Chia flour (X_1), Quinoa flour (X_2) and Amaranth flour (X_3) on bread calorie value

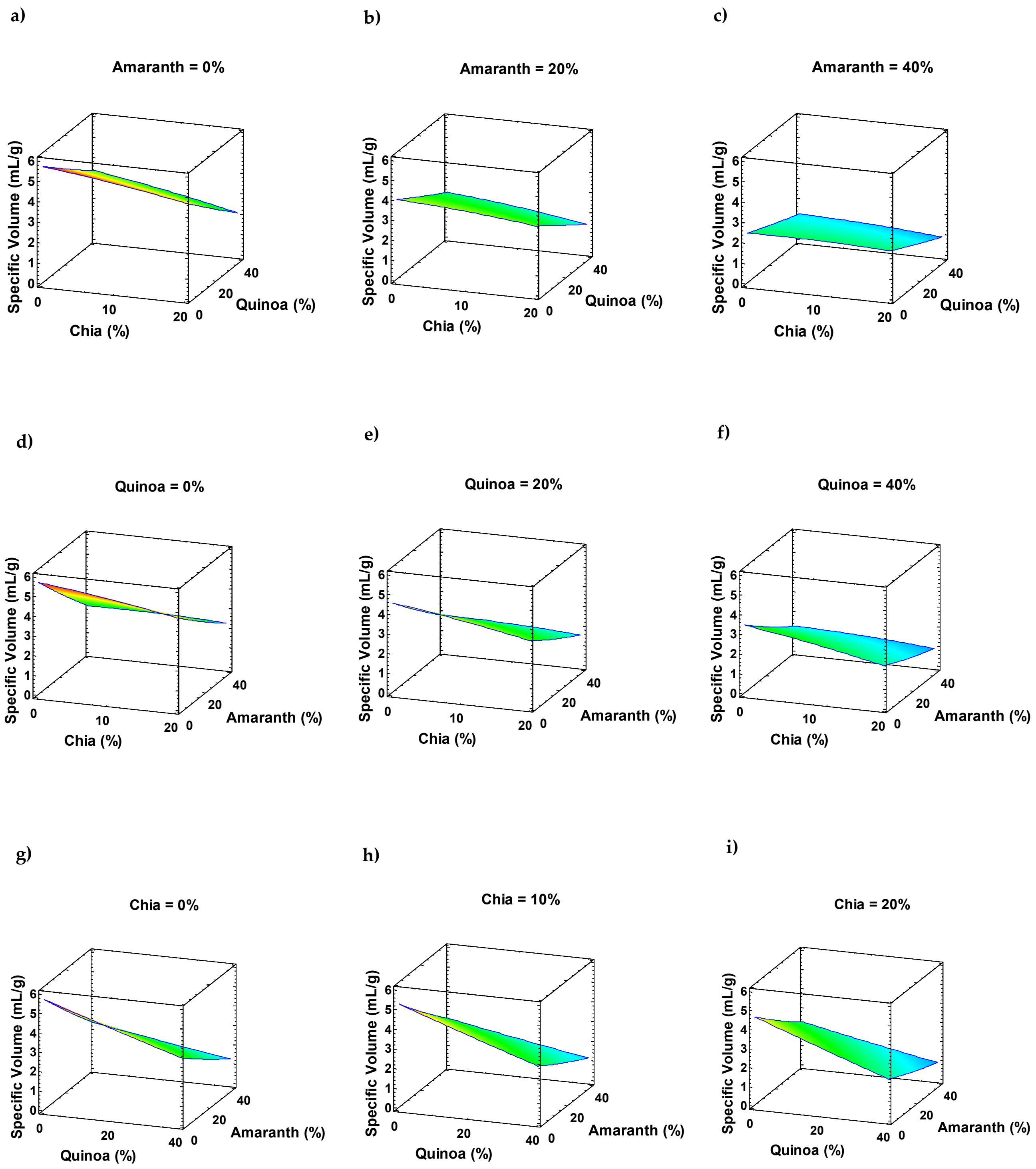

Figure S3. Influence of interaction between the factors Chia flour (X_1), Quinoa flour (X_2) and Amaranth flour (X_3) on bread specific volume

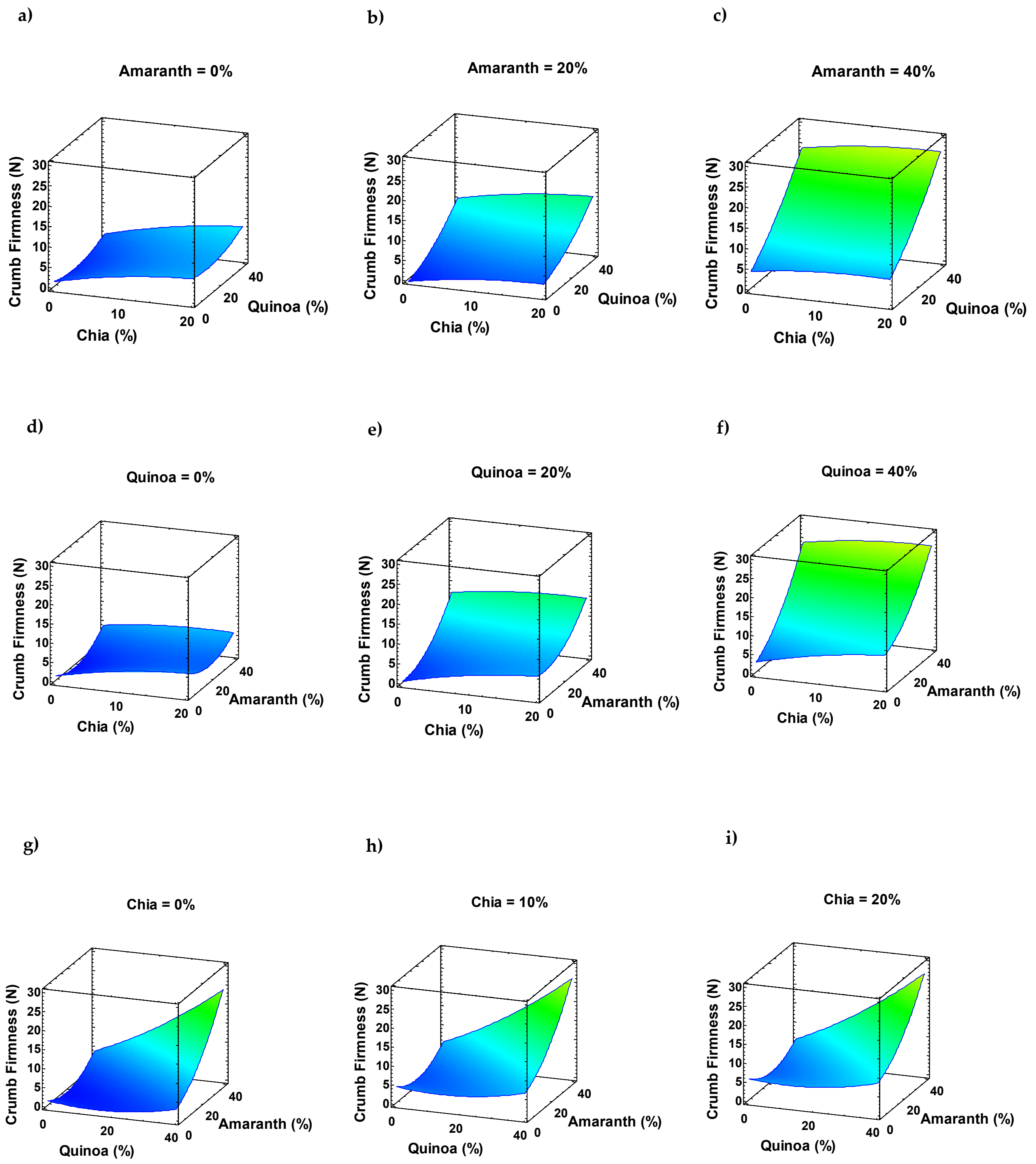

Figure S4. Influence of interaction between the factors Chia flour (X_1), Quinoa flour (X_2) and Amaranth flour (X_3) on crumb firmness

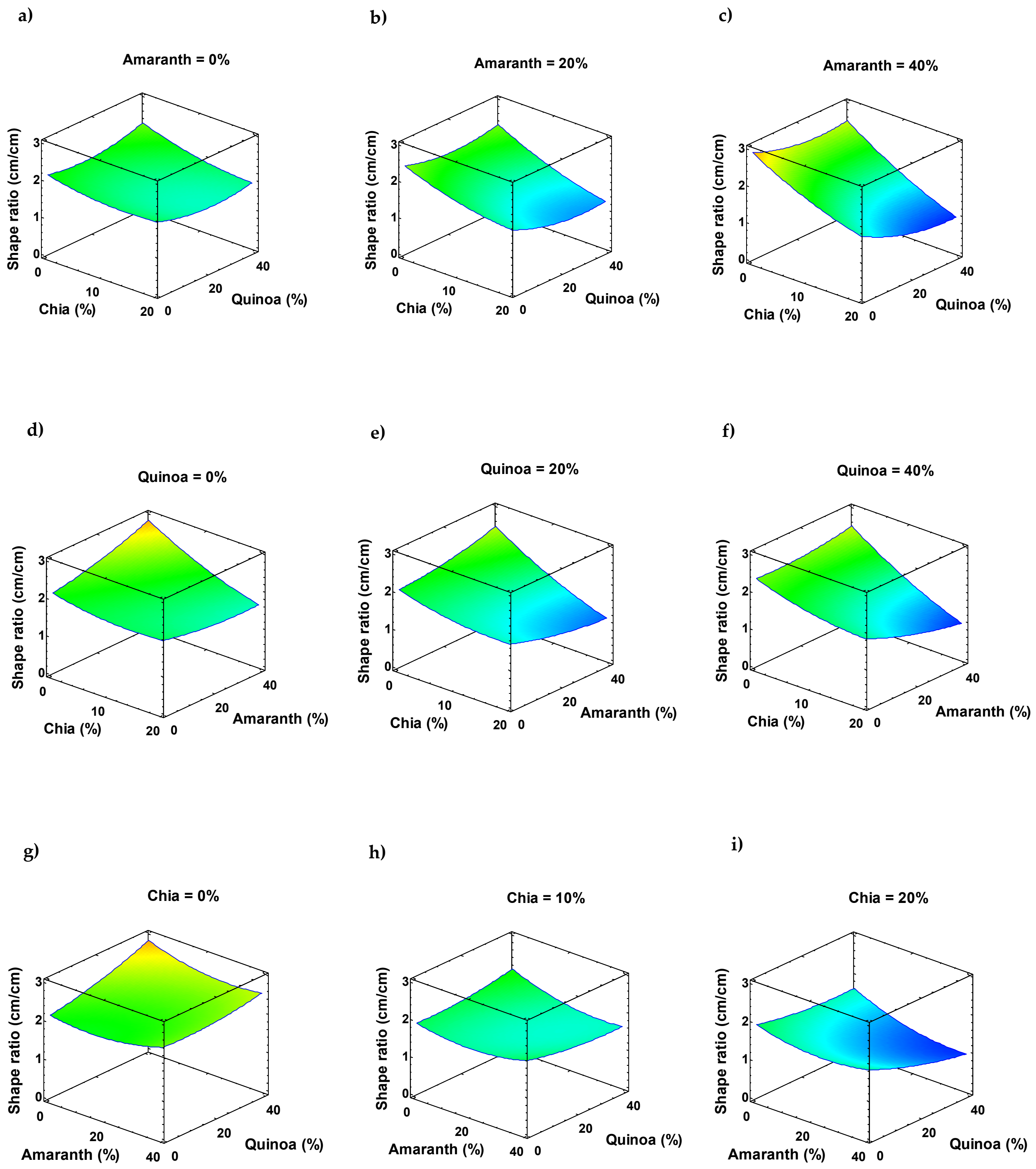

Figure S5. Influence of interaction between the factors Chia flour (X_1), Quinoa flour (X_2) and Amaranth flour (X_3) on shape ratio

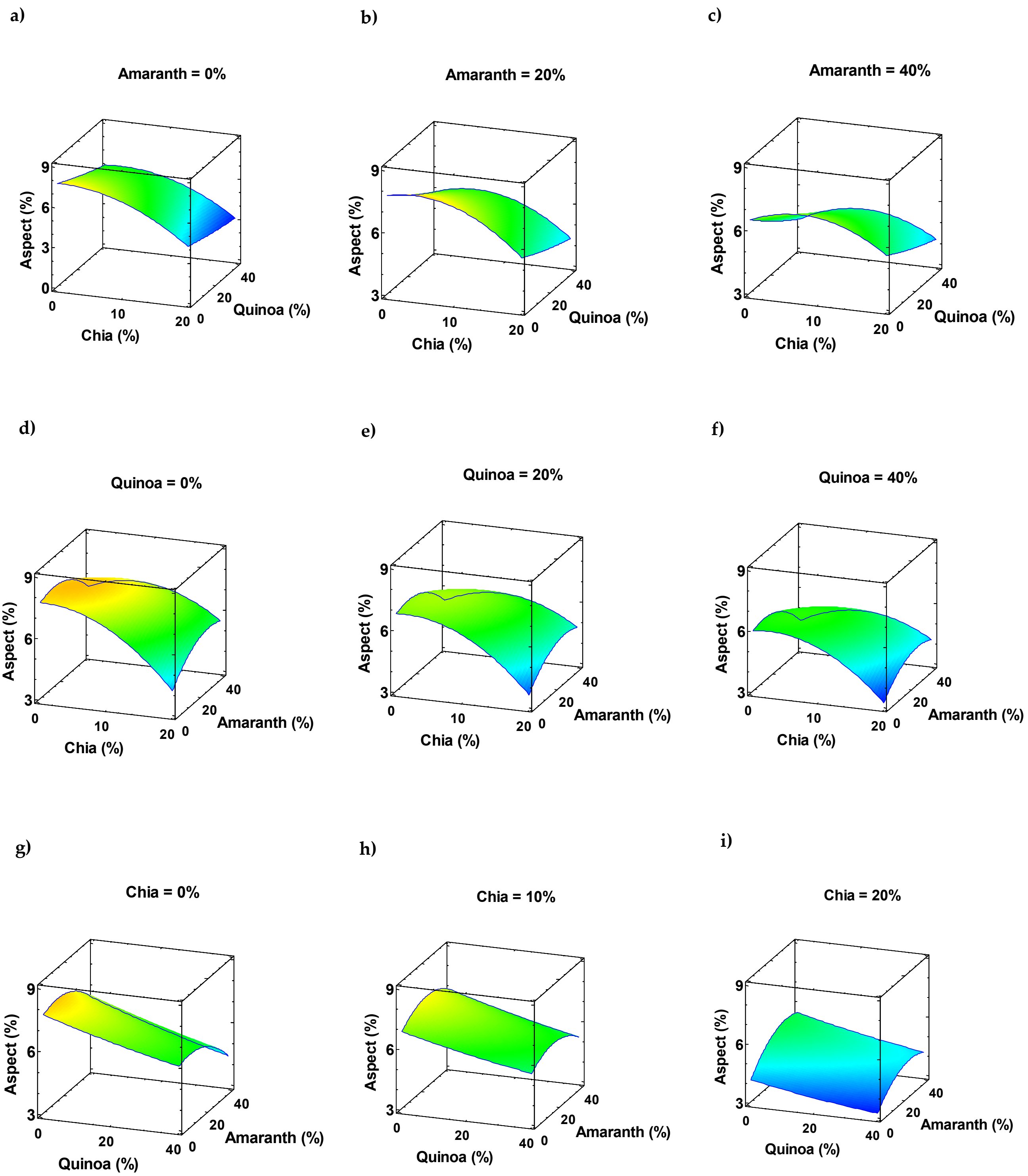

Figure S6. Influence of interaction between the factors Chia flour (X_1), Quinoa flour (X_2) and Amaranth flour (X_3) on sensorial evaluation, aspect
